# The Impact of Distance and Income on Pediatric Solid Extracranial Tumors: A Report From CYP‐C

**DOI:** 10.1002/cam4.71504

**Published:** 2025-12-26

**Authors:** Olivia Piccolo, Kara Matheson, Stacey Marjerrison, Ketan Kulkarni, Rodrigo Romao, Craig Erker

**Affiliations:** ^1^ Faculty of Medicine Dalhousie University Halifax Nova Scotia Canada; ^2^ Research Methods Unit Nova Scotia Health Halifax Nova Scotia Canada; ^3^ Faculty of Health Sciences McMaster University Hamilton Ontario Canada; ^4^ Division of Hematology/Oncology IWK Health Centre Halifax Nova Scotia Canada; ^5^ Division of Pediatric Urology/General Surgery SickKids Hospital Toronto Ontario Canada

**Keywords:** epidemiology, health care access, health equity, hepatoblastoma, neuroblastoma, rhabdomyosarcoma, socio‐economic status

## Abstract

**Background:**

The impact of social determinants of health (SDoH) on survival outcomes is unclear in the universal Canadian health care system. We investigated the impact of distance to treatment center and income quintile on survival outcomes in pediatric extracranial solid tumors in Canada.

**Methods:**

Children < 15 years old diagnosed with 7 common solid extracranial tumors from 2001 to 2020 were included using the Cancer in Young People in Canada (CYP‐C) data tool. We used logistic regression to examine the association of income quintile and distance on cancer outcomes. We used Cox proportional hazard models to examine associations with time‐to‐event outcomes (OS) and Fine–Gray competing risk regression (recurrence) adjusting for metastasis, age, region, and tumor location.

**Results:**

The cohort included 3969 patients. Median age was 3.6 years (IQR: 1.3–8.9); 48.7% were female. Tumor diagnosis: 34% neuroblastoma, 21% Wilms tumor, 13% rhabdomyosarcoma, 11% osteosarcoma, 8% Ewing sarcoma, 7% hepatoblastoma, and 6% germ cell tumors. On multivariable analysis, income quintile and distance did not significantly or consistently impact survival across all tumors. In rhabdomyosarcoma, the second lowest income quintile had inferior survival compared to the highest income quintile (*p* = 0.0264, HR 1.91, 95% CI 1.08, 3.37). In neuroblastoma, the lowest income quintile had inferior survival compared to the highest (*p* = 0.0052, HR 1.82, 95% CI 1.20, 2.77). Patients diagnosed with hepatoblastoma living > 500 km from a pediatric treatment facility had inferior OS compared to those within 50 km (*p* = 0.0065, HR 3.29, 95% CI 1.40, 7.79).

**Conclusion:**

Overall, distance and income did not show a consistent significant impact on survival outcomes for children with extracranial solid tumors.

## Abstract 2

In this national analysis of Canadian children with solid extracranial tumors, our results do not demonstrate a consistent relationship between socioeconomic status or distance and survival outcomes across all solid tumors.

## 1 Introduction

Childhood cancer is the leading cause of disease‐related mortality in children in Canada [1]. Solid tumors in children are a common cause of childhood death, can persist undetected and often present in late stages [2, 3]. In addition to the known prognostic features associated with solid malignancies, the social determinants of health (SDoH) are critical conditions that shape an individual's healthcare access and have the potential to impact survival. SDoH and their impact on survival outcomes are poorly described in pediatric solid extracranial tumors.

In pediatric patients with head and neck cancer, lower socioeconomic status (SES) has been associated with inferior survival in China, Canada, and the USA [4, 5, 6]. Existing literature in pediatric leukemia and lymphoma suggests that lower SES is associated with worse overall and event‐free survival in the USA [7]. Mechanisms underlying the association between higher SES and superior cancer outcomes are postulated to include earlier treatment‐seeking in the disease process [8], better access to clinical trials and supportive care [9, 10], adherence to therapy, and ability to relocate closer to a tertiary care center if needed [9]. This evidence from thyroid tumors, leukemia, and lymphoma suggests the potential impact of SES on survival outcomes, but this has not been investigated in all solid tumors in children [5, 8, 10].

Income quintile for families in Canada has previously been used as a surrogate for economic stability and wellbeing [11, 12, 13]. While the studies mentioned above highlight a potential link between lower SES and inferior survival in children with cancer, more recent Canadian studies in leukemia and lymphoma demonstrate that these differences may be mitigated by our universal healthcare system [13]. This warrants further evaluation into the impact of income quintile on survival in other pediatric tumor types. Healthcare in Canada, including hospital and physician services to provide cancer care, is delivered through provincial universal single‐payer insurance systems funded by the provincial and federal governments. Some costs, like physical therapy, psychosocial services, and outpatient medications, are accounted for by a mixture of publicly funded programs and private insurance—although not all children have access. Also, distance to treatment center has been used as a proxy measure for healthcare access and has previously been associated with worsening outcomes in pediatric leukemia [14].

The purpose of this work is to examine the impact of neighborhood median household income quintile and distance to treatment center on survival outcomes in children with solid extracranial tumors.

## 2 Methods

### 2.1 Study Design and Population

This was a retrospective cohort study of children aged less than 15 years diagnosed with one of seven solid extracranial tumors between January 1, 2001 and December 31, 2019 in Canada. The diagnoses included Wilms' tumor, neuroblastoma, rhabdomyosarcoma, hepatoblastoma, osteosarcoma, Ewing Sarcoma, and malignant gonadal germ cell tumors. All patients diagnosed at one of the 16 pediatric hematology, oncology, and stem cell transplant programs in Canada were included, which encompasses all pediatric patients diagnosed with one of those seven extracranial solid tumors from 2001 to 2019.

### 2.2 Data Source

Data were provided by the Cancer in Young People in Canada (CYP‐C) Data Tool—a Canadian population‐based surveillance system for pediatric oncology patient information. The database collaborates with the Public Health Agency of Canada, the Canadian Partnership Against Cancer, and the C17 Council network of all Canadian pediatric oncology centers [15]. CYP‐C collects and maintains data on all children diagnosed with malignancy between the ages of 0 and 14 years and treated at one of the 16 pediatric hematology, oncology, and stem cell transplant programs in Canada [1]. In Ontario, data are primarily collected by the Pediatric Oncology Group of Ontario (POGO), which maintains its own registry of cancer cases diagnosed or treated in its 5 pediatric centers (POGONIS–POGO Network Information System). Outside Ontario, clinically relevant data on new cancer diagnoses and treatment are gathered from patient medical charts at pediatric oncology centers and entered directly into CYP‐C's electronic database. CYP‐C data includes demographic variables, diagnostic details, time to diagnosis and treatment, income quintile, distance to pediatric oncology center, outcomes such as relapse, second malignancy and death for 5 years after diagnosis [15]. The terms “pediatric oncology centre,” “treatment centre,” and “reporting centre” are used interchangeably. Data for this study were extracted on October 4 2023. A waiver of consent was granted by the IWK Institutional Review Board for the purpose of this study.

### 2.3 Outcomes

The primary outcome of interest was overall survival (OS) for neuroblastoma, rhabdomyosarcoma, osteosarcoma, Ewing sarcoma, and hepatoblastoma. OS was defined as the length of time from the date of diagnosis to death from any cause. OS was the preferred outcome measure due to its important clinical relevance and robustness in the CYP‐C registry. However, in Wilms tumor and malignant gonadal germ cell tumors, progression/relapse, which was defined as “recurrence,” with death as a competing risk was the primary outcome of interest. In these tumors, OS was high and there were not enough events for consideration; therefore, event‐free survival (EFS) with death as a competing risk was used. Recurrence was defined as the length of time from the date of diagnosis to relapse or progression, whichever occurred first. EFS with death as a competing risk was not the primary outcome measure in tumors where there were sufficient events to analyze OS, which was preferred. Due to differences in tumor biology and treatment, we examined each diagnosis separately to account for tumor‐specific survival differences.

### 2.4 Primary Predictors of Research Hypothesis

Drawing from previous studies, neighborhood income quintiles were used as a surrogate measure for economic stability, and therefore socioeconomic status (SES) [11, 12, 13]. This variable was based on each child's residential postal code at the time of diagnosis, and any patients that were missing postal code were excluded. Neighborhood income quintiles were defined according to Statistics Canada methods whereby Dissemination Areas (DAs) average household income is ranked into quintiles ranging from least (Quintile 1) to most affluent (Quintile 5), a measure that adjusts for household size, cost of living, and regional differences [16]. Each DA encompasses a population target of 400–700 people within the designated postal code area. These data are available via Statistics Canada's Postal Code Conversion File plus (PCCF+) software Version 5K.

Distance to reporting center was used as a surrogate measure for access to health care. This was a derived variable of the distance from the patient's home address to their reporting center, based on the difference (in kms) between the reporting centre latitude and longitude and the patient's place of residence latitude and longitude. We further categorized distance to reporting centre into quartiles: (a) < 50 km, (b) 50 to < 200 km, (c) 200 to < 500 km, and (d) > 500 km. These distance cut‐offs were chosen as they were used in previous literature examining distance to reporting center in Canada [17]. Patients with an unknown location of residence at diagnosis or missing the variable of distance to reporting center were excluded.

### 2.5 Covariates

Demographic features included in our models were age at diagnosis (< 4 years, 4 to < 9 years, and > 9 years), biologic sex, year of diagnosis in 5‐year increments, and geographic region of Canada. We examined the dataset and selected these age cut‐offs to encompass the distribution of age in different tumor diagnoses. Diagnostic information included in our models were tumor primary sites and metastasis at diagnosis. Tumor primary sites were provided by CYP‐C, and this data was categorized into distinct anatomical regions of the body when appropriate including (a) abdomen and retroperitoneum, (b) soft tissue, (c) bone, (d) head and neck, (e) genital region, (f) thorax and mediastinum, and (g) other. The rationale to include tumor primary sites was to account for diagnoses where certain anatomical sites might have more favorable survival outcomes, such as in rhabdomyosarcoma and neuroblastoma. Tumor location was only accounted for in tumors where location varied. Provincial‐level data was collected and analyzed but is censored from display due to CYP‐C policy framework. Patients with intracranial primary tumor sites were excluded. We did not include race or ethnicity as covariates in our analysis due to significant missing data in the reporting of these variables in CYP‐C database. We did not include cause of death as a covariate in our analysis due to significant differences in attribution of causes of death among the different reporting centers in CYP‐C database.

### 2.6 Statistical Analysis

Descriptive statistics were reported as counts and percentages for categorical variables, means + standard deviation for normally distributed continuous variables, and medians and interquartile ranges for non–normally distributed continuous variables. Box‐plots were used to display the distribution of continuous variables. OS was analyzed using Kaplan–Meier plots and log‐rank tests to compare the survival distribution between a priori selected patient demographic variables: income quintile, region of Canada, metastasis at diagnosis, tumor location, age, and distance to reporting center.

Univariable and multivariable Cox proportional hazard models were used to generate hazard ratios (HRs) and 95% confidence intervals to compare rates of survival separately for each primary tumor site including neuroblastoma, rhabdomyosarcoma, hepatoblastoma, osteosarcoma, and Ewing Sarcoma. Models were separated into specific disease groups due to the different associations between outcomes and covariates due to biologic distinctions of disease subtypes. For the multivariable model, covariates included were discussed a priori and selected based on clinical knowledge, with distance and income quintile included in each model. Children not experiencing an event were censored on the date of last follow‐up—measured as their last recorded contact with the healthcare system. The proportional hazards assumption was tested using the Kolmogorov‐type supremum test and exploring time by covariate interactions. Interactions between the primary outcome variables income quintile and distance to reporting center were tested for statistical significance. A competing risk analysis was performed for the outcome of recurrence with mortality as a competing risk. The cumulative incidence function was compared between groups using Gray's method. Differences in recurrence were compared using a Fine–Gray sub‐distribution hazard model for competing risks regression. Sub‐distribution hazard ratio (SHR) and 95% confidence intervals are reported for primary tumor site including Wilm's tumor and malignant gonadal Germ cell tumors. All analyses were performed on complete cases for all variables using SAS STAT 15.1 version 9.4 (SAS Institute, Cary, N.C., USA). A two‐sided *p* value of < 0.05 was the threshold for statistical significance unless otherwise stated.

### 2.7 Funding and Ethics Approval

Research Ethics Board approval was granted by the IWK (file no. 1029288) on July 5th, 2023.

## 3 Results

### 3.1 Study Cohort

**FIGURE 1:**
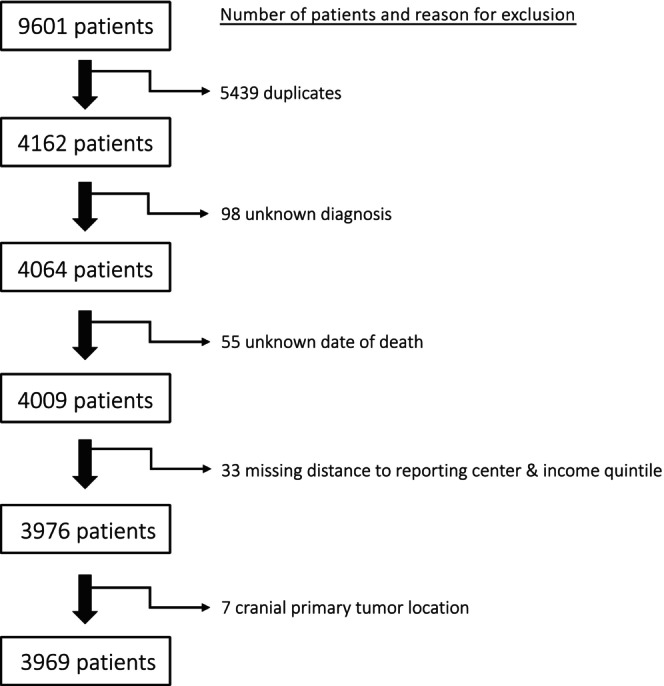
Flow chart outlining the data cleaning and exclusion process for the cohort.

**FIGURE 2:**
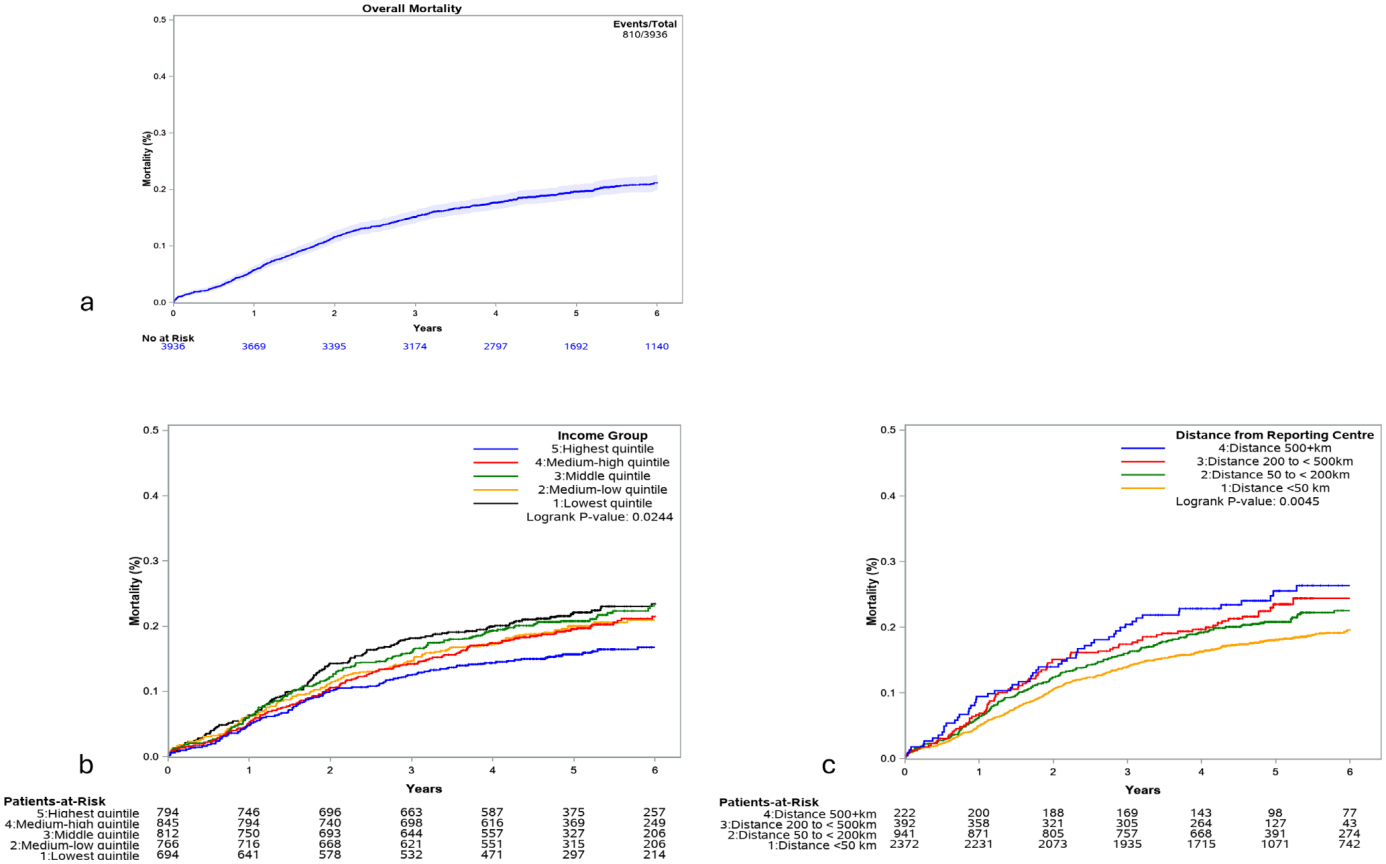
Kaplan–Meier survival plots for (a) overall mortality, (b) income, and (c) distance from reporting center for all solid tumor diagnoses: neuroblastoma, rhabdomyosarcoma, osteosarcoma, Ewing sarcoma, hepatoblastoma, Wilms tumor, and Germ cell tumor.

We received data on 4162 pediatric patients presenting with Wilms' tumor, neuroblastoma, rhabdomyosarcoma, hepatoblastoma, osteosarcoma, Ewing sarcoma, and germ cell tumors between 2001 and 2020. Our selection process excluded patients with unknown diagnosis (*n* = 98), patients with an unknown date of death (*n* = 55), patients missing distance and income variables (*n* = 33), and patients with cranial primary tumors (*n* = 7) (Figure 1). The characteristics of the remaining 3969 children are presented in Table 1. Kaplan–Meier survival plots for overall mortality, income, and distance for all solid tumors are presented in Figure 2. There were significant differences in overall survival probabilities among all tumors for our study timeline with respect to income quintiles (Logrank *p* = 0.0245, Figure 2b) and distance from treatment facility (Logrank *p* = 0.0045, Figure 2c). Univariate analysis for all tumor types is shown in Table 2, but all results described pertain to multivariable testing.

**TABLE 1:** Demographic and tumor‐specific characteristics of the study population (*n* = 3969).

Tumor diagnosis	Neuroblastoma (*n* = 1349)	Rhabdomyosarcoma (*n* = 503)	Osteosarcoma (*n* = 432)	Ewing's sarcoma (*n* = 299)	Hepatoblastoma (*n* = 266)	Wilms tumor (*n* = 823)	Malignant gonadal germ cell tumor (*n* = 225)
Demographic variables							
Age							
< 4 years	1067 (79.4%)	187 (37.2%)	8 (1.9%)	27 (9.0%)	225 (84.6%)	511 (62.1%)	52 (23.1%)
4 to < 9 years	235 (17.5%)	173 (34.4%)	68 (15.7%)	66 (22.1%)	26 (9.8%)	269 (32.7%)	27 (12.0%)
> 9 years	41 (3.1%)	143 (28.4%)	356 (82.4%)	206 (68.9%)	15 (5.6%)	43 (5.2%)	146 (64.9%)
Mean (SD)	2.4 (2.6)	6.5 (4.4)	11.9 (2.9)	10.3 (3.8)	2.5 (2.8)	3.7 (2.6)	9.9 (5.3)
Median (IQR)	1.6 (1, 3)	5.4 (3, 10)	12.7 (10, 14)	11.0 (8, 13)	1.6 (1, 3)	3.2 (2, 5)	11.8 (6, 14)
Gender							
Female	613 (45.4%)	227 (45.1%)	215 (49.9%)	146 (48.8%)	110 (41.4%)	441 (53.6%)	134 (59.6%)
Male	736 (54.6%)	276 (54.9%)	216 (50.1%)	153 (51.2%)	156 (58.6%)	382 (46.4%)	91 (40.4%)
Time from first visit to plan start date (days)							
Mean (SD)	37 (94)	37 (76)	34 (46.6)	48 (81)	31 (75)	24 (62)	20 (32)
Median (IQR)	16 (7, 35)	21 (10, 37)	21 (14, 35)	24 (16, 44)	12 (7, 25)	10 (5, 21)	9 (4, 22)
Diagnostic variables							
Primary tumor region							
Abdominal	883 (65.5%)	44 (8.7%)	0 (0.0%)	< 5 (0.3%)	266 (100.0%)	< 823 (< 100%)	0 (0.0%)
Thorax/mediastinum	196 (14.5%)	15 (3.0%)	0 (0.0%)	7 (2.4%)	0 (0.0%)	0 (0.0%)	0 (0.0%)
Soft tissue	203 (15.0%)	170 (33.8%)	0 (0.0%)	< 5 (0.7%)	0 (0.0%)	0 (0.0%)	0 (0.0%)
Bone	23 (1.7%)	13 (2.6%)	427 (99.1%)	281 (94.6%)	0 (0.0%)	0 (0.0%)	0 (0.0%)
Head and neck	13 (1.0%)	127 (25.2%)	< 5 (< 1.0%–0.7%)	< 5 (1.0%)	0 (0.0%)	0 (0.0%)	0 (0.0%)
Genital region	21 (1.6%)	127 (25.2%)	< 5 (< 0.5%)	< 5 (0.7%)	0 (0.0%)	0 (0.0%)	225 (100.0%)
Other	10 (0.7%)	7 (1.4%)	0 (0.0%)	< 5 (0.3%)	0 (0.0%)	< 5 (< 0.5%)	0 (0.0%)
Metastasis at diagnosis							
Yes	728 (54.0%)	167 (33.2%)	104 (24.1%)	112 (37.5%)	58 (21.8%)	246 (29.9%)	52 (23.1%)
No	596 (44.2%)	323 (64.2%)	321 (74.3%)	< 185 (< 61.5%)	194 (72.9%)	554 (67.3%)	< 175 (< 76.0%)
Missing	25 (1.9%)	13 (2.6%)	7 (1.6%)	< 5 (< 1.5%)	14 (5.3%)	23 (2.8%)	< 5 (< 1.5%)
Recurrence							
Yes	367 (27.2%)	184 (36.6%)	163 (37.7%)	103 (34.4%)	64 (24.1%)	150 (18.2%)	31 (13.8%)
No	982 (72.8%)	319 (63.4%)	269 (62.3%)	196 (65.6%)	202 (75.9%)	673 (81.8%)	194 (86.2%)
Economic stability variable							
Income quintile							
1 (lowest)	247 (18.3%)	71 (14.1%)	64 (14.8%)	42 (14.0%)	65 (24.4%)	154 (18.7%)	42 (18.7%)
2	256 (19.0%)	99 (19.7%)	91 (21.1%)	55 (18.4%)	59 (22.2%)	152 (18.5%)	50 (22.2%)
3	283 (21.0%)	106 (21.1%)	96 (22.2%)	71 (23.7%)	38 (14.3%)	164 (19.9%)	47 (20.9%)
4	295 (21.9%)	117 (23.3%)	89 (20.6%)	73 (24.4%)	66 (24.8%)	159 (19.3%)	40 (17.8%)
5 (highest)	260 (19.3%)	110 (21.9%)	82 (19.0%)	57 (19.1%)	37 (13.9%)	189 (23.0%)	46 (20.4%)
Neighborhood and built environment							
Distance to reporting center							
< 50 km	841 (62.3%)	313 (62.2%)	253 (59.7%)	176 (58.9%)	131 (49.2%)	497 (60.5%)	138 (61.3%)
50 to < 200 km	306 (22.7%)	125 (24.9%)	104 (24.5%)	73 (24.4%)	68 (25.6%)	194 (23.6%)	62 (27.6%)
200 to < 500 km	116 (8.6%)	40 (8.0%)	43 (10.1%)	34 (11.4%)	41 (15.4%)	94 (11.4%)	< 25 (< 10.0%)
> 500 km	86 (6.4%)	25 (5.0%)	24 (5.7%)	16 (5.4%)	26 (9.8%)	37 (4.5%)	< 5 (< 2.0%)
Mean (SD)	157.6 (427)	111.0 (278)	140.5 (334)	154.5 (377)	210.5 (451)	117.4 (257)	81.6 (130)
Median (IQR)	30.1 (12, 99)	27.2 (11, 87)	30.7 (13,140)	36.2 (15,108)	51.9 (17, 201)	29.7 (12, 115)	31.0 (12, 89)

Abbreviations: IQR, interquartile range; km, kilometers; SD, standard deviation.

**TABLE 2:** Univariate predictors of survival.

Diagnosis	Neuroblastoma (*n* = 1349)		Rhabdomyosarcoma (*n* = 503)		Osteosarcoma (*n* = 432)		Ewing sarcoma (*n* = 299)		Hepatoblastoma (*n* = 266)		Wilms tumor (*n* = 823)		Germ cell tumor (*n* = 225)	
Covariates	Overall survival (OS)										Recurrence			
	HR (95% CI)	*p*	HR (95% CI)	*p*	HR (95% CI)	*p*	HR (95% CI)	*p*	HR (95% CI)	*p*	HR (95% CI)	*p*	HR (95% CI)	*p*
Age (vs. < 4 years)														
4 to < 9 years	1.98 (1.52, 2.59)	< 0.001	0.97 (0.64, 1.45)	0.863	1.06 (0.25, 4.53)	0.933	1.51 (0.42, 5.42)	0.526	0.76 (0.27, 2.1)	0.593	1.31 (0.93, 1.83)	0.118	0.74 (0.14, 3.84)	0.723
> 9 years	1.89 (1.08, 3.32)	0.027	1.65 (1.13, 2.41)	0.010	1.32 (0.33, 5.33)	0.699	3.99 (1.26, 12.6)	0.018	1.78 (0.76, 4.18)	0.183	0.96 (0.45, 2.05)	0.917	1.65 (0.62, 4.37)	0.318
Gender														
Female vs. male	0.71 (0.56, 0.91)	0.006	1.15 (0.84, 1.58)	0.392	0.66 (0.48, 0.91)	0.011	0.72 (0.48, 1.11)	0.134	1.85 (1.09, 3.14)	0.024	1.23 (0.89, 1.71)	0.212	1.59 (0.73, 3.47)	0.241
Metastasis at diagnosis														
Yes vs. no	7.51 (5.11, 11.1)	< 0.001	4.29 (3.06, 5.98)	< 0.001	3.35 (2.42, 4.64)	< 0.001	3.18 (2.07, 4.90)	< 0.001	3.20 (1.85, 5.57)	< 0.001	1.96 (1.42, 2.72)	< 0.001	1.46 (0.67, 3.15)	0.34
Income quintile (vs. highest)														
Lowest (1)	1.47 (0.99, 2.2)	0.058	1.73 (0.98, 3.07)	0.059	1.49 (0.88, 2.52)	0.143	1.32 (0.62, 2.81)	0.472	0.88 (0.4, 1.94)	0.747	0.87 (0.52, 1.46)	0.602	0.95 (0.32, 2.76)	0.917
Medium‐low (2)	1.29 (0.86, 1.94)	0.220	1.58 (0.92, 2.70)	0.099	1.22 (0.74, 2.02)	0.436	1.13 (0.55, 2.34)	0.743	0.65 (0.28, 1.54)	0.330	1.3 (0.82, 2.08)	0.277	0.52 (0.16, 2.08)	0.278
Middle (3)	1.39 (0.94, 2.07)	0.099	1.71 (1.0,2.90)	0.048	1.13 (0.69, 1.86)	0.624	1.14 (0.58, 2.27)	0.700	0.55 (0.2, 1.52)	0.251	0.85 (0.51, 1.4)	0.519	0.86 (0.29, 2.56)	0.792
Medium‐high (4)	1.31 (0.89, 1.94)	0.177	1.73 (1.04, 2.89)	0.036	0.81 (0.47, 1.39)	0.443	1.54 (0.8, 2.94)	0.196	0.57 (0.24, 1.35)	0.204	0.81 (0.48, 1.36)	0.416	1.2 (0.43, 3.39)	0.730
Distance (vs. < 50 km)														
50 to < 200 km	1.02 (0.76, 1.38)	0.890	1.28 (0.89, 1.84)	0.192	1.33 (0.93, 1.9)	0.124	0.96 (0.57, 1.62)	0.888	1.19 (0.59, 2.37)	0.631	1.29 (0.89, 1.87)	0.188	1.12 (0.51, 2.46)	0.787
200 to < 500 km	1.33 (0.87, 2.04)	0.181	1.48 (0.86, 2.56)	0.160	1.02 (0.59, 1.77)	0.933	1.68 (0.93, 3.03)	0.087	1.46 (0.67, 3.18)	0.347	1.11 (0.66, 1.88)	0.698	1.11 (0.33, 3.79)	0.867
> 500 km	1.46 (0.96, 2.22)	0.080	1.20 (0.58, 2.48)	0.619	0.82 (0.38, 1.78)	0.618	0.72 (0.26, 1.98)	0.528	3.35 (1.64, 6.83)	< 0.001	0.75 (0.32, 1.79)	0.518	ID	ID
Year of diagnosis														
Per 5‐year increase	0.87 (0.77, 0.98)	0.020	1.12 (0.96, 1.30)	0.153	1.10 (0.94, 1.27)	0.2341	0.77 (0.62, 0.94)	0.011	0.94 (0.74, 1.20)	0.6189	0.82 (0.71, 0.96)	0.012	0.92 (0.67, 1.24)	0.568

Abbreviations: CI, confidence interval; HR, hazard ratio; ID, insufficient data; km, kilometers.

### 3.2 Multivariable Analysis—Overall Survival

#### 3.2.1 Neuroblastoma

The 5‐year OS rate for patients with neuroblastoma was 81.6% (95% CI 79.5, 83.8%). Compared to other tumors, the incidence of metastasis at diagnosis was highest in neuroblastoma (54%). Most neuroblastoma primary tumors were found in the abdomen and retroperitoneum (66%). Patients with the lowest income quintile had inferior OS compared to patients with the highest income quintile (HR 1.82 [95% CI 1.20, 2.77], *p* = 0.0052). Otherwise, there were no additional significant findings regarding income quintiles. Distance to treatment facility did not significantly impact OS in patients with neuroblastoma. Other significant findings demonstrate that compared to children age < 4 years old, children 4 to 9 years old (HR 1.96 [95% CI 1.50, 2.61], *p* < 0.0001) and > 9 years old (HR 2.57 [95% CI 1.42, 4.63], *p* = 0.0017; Table 3) had inferior OS. Metastasis was associated with increased mortality (HR 6.67 [95% CI 4.47, 9.94], *p* < 0.0001). Patients with tumors in their abdomen/retroperitoneum had inferior OS than patients with tumors in their thorax/mediastinum (HR 2.12 [95% CI 1.26, 3.55], *p* = 0.0044). Female patients had decreased risk of death compared to males with neuroblastoma (HR 0.73 [95% CI 0.57, 0.95], *p* = 0.017). Survival rate improved over time (HR 0.85 [95% CI 0.75, 0.96], *p* = 0.0096, expressed per 5‐year increase).

**TABLE 3:** Multivariable predictors of survival.

Diagnosis	Neuroblastoma (*n* = 1349)		Rhabdomyosarcoma (*n* = 503)		Osteosarcoma (*n* = 432)		Ewing sarcoma (*n* = 299)		Hepatoblastoma (*n* = 266)		Wilms tumor (*n* = 823)		Germ cell tumor (*n* = 225)	
	Overall survival (OS)										Event‐free survival (EFS)			
	HR (95% CI)	*p*	HR (95% CI)	*p*	HR (95% CI)	*p*	HR (95% CI)	*p*	HR (95% CI)	*p*	HR (95% CI)	*p*	HR (95% CI)	*p*
Age (vs. < 4 years)														
4 to < 9 years	1.98 (1.50,2.61)	< 0.001	0.91 (0.60, 1.40)	0.6680	0.61 (0.14, 2.67)	0.5088	1.45 (0.39, 5.42)	0.5836	0.52 (0.12, 2.31)	0.3900	1.05 (0.72, 1.52)	0.8015	0.33 (0.05, 2.41)	0.2742
> 9 years	2.57 (1.42, 4.63)	0.0017	1.23 (0.81, 1.87)	0.3226	0.74 (0.18, 3.03)	0.6696	3.00 (0.91, 9.89)	0.0707	1.32 (0.52, 3.23)	0.5558	0.69 (0.30, 1.59)	0.3793	1.07 (0.34, 3.39)	0.9132
Primary tumor region														
Abdomen/retroperitoneum	2.12 (1.26, 3.55)	0.0044	2.13 (1.03, 4.38)	0.0408										
Other			3.01 (1.52, 5.97)	0.0016										
Soft tissue	1.74 (0.97, 3.13)	0.0646	2.32 (1.36, 3.95)	0.0020										
Head and neck			2.28 (1.27, 4.09)	0.0056										
Metastasis at diagnosis														
Yes vs. no	6.67 (4.47, 9.94)	< 0.001	3.95 (2.76, 5.65)	< 0.001	3.37 (2.38, 4.78)	< 0.001	3.35 (2.11, 5.32)	< 0.001	4.41 (2.38, 8.16)	< 0.001	2.05 (1.43, 2.96)	0.0001	1.35 (0.55, 3.28)	0.5146
Income quintile (vs. highest)														
Lowest (1)	1.82 (1.20, 2.77)	0.0052	1.78 (0.98, 3.22)	0.0591	1.51 (0.87, 2.61)	0.1397	1.02 (0.46, 2.26)	0.9663	1.09 (0.41, 2.86)	0.8676	0.92 (0.55, 1.55)	0.7488	1.25 (0.39,4.06)	0.7056
Medium‐low (2)	1.26 (0.82, 1.93)	0.2847	1.91 (1.08, 3.37)	0.0264	1.06 (0.63, 1.78)	0.8333	0.94 (0.43, 2.02)	0.8643	0.92 (0.33, 2.60)	0.8797	1.26 (0.77, 2.04)	0.3571	0.46 (0.14, 1.56)	0.2136
Middle (3)	1.42 (0.94, 2.13)	0.0921	1.42 (0.81, 2.49)	0.2229	1.03 (0.61, 1.71)	0.9208	1.13 (0.54, 2.34)	0.7431	0.59 (0.18, 1.93)	0.3837	0.81 (0.48, 1.36)	0.4296	0.75 (0.22,2.49)	0.6343
Medium‐high (4)	1.24 (0.82, 1.87)	0.3006	1.67 (0.97, 2.86)	0.0627	0.79 (0.45, 1.36)	0.3857	1.22 (0.60, 2.48)	0.5831	0.94 (0.36, 2.61)	0.9580	1.07 (0.70, 1.62)	0.4805	1.28 (0.44, 3.72)	0.6475
Distance (vs. < 50 km)														
50 to < 200 km	1.01 (0.74, 1.37)	0.9960	1.12 (0.76, 1.65)	0.5690	1.56 (1.04, 2.34)	0.0299	1.13 (0.64, 1.97)	0.6766	0.82 (0.36, 1.89)	0.6481	1.07 (0.70, 1.62)	0.7581	1.22 (0.53, 2.81)	0.6322
200 to < 500 km	1.11 (0.69, 1.79)	0.6777	1.08 (0.59, 1.98)	0.8010	1.51 (0.82, 2.79)	0.1863	1.66 (0.84, 3.26)	0.1458	1.07 (0.44, 2.61)	0.8761	0.80 (0.46, 1.39)	0.4276	0.84 (0.21, 3.42)	0.8069
> 500 km	0.96 (0.61, 1.52)	0.8691	0.93 (0.43, 2.02)	0.8552	0.68 (0.29, 1.58)	0.3660	0.99 (0.34, 2.90)	0.9793	3.29 (1.40, 7.79)	0.0065	0.52 (0.20, 1.33)	0.1726	ID	ID
Gender														
Female vs. male	0.73 (0.57, 0.95)	0.0170	0.92 (0.65, 1.30)	0.6401	0.65 (0.46, 0.93)	0.0167	0.91 (0.57, 1.44)	0.6850	1.85 (0.99, 3.44)	0.0514	1.23 (0.88, 1.74)	0.2298	1.63 (0.65, 4.07)	0.2936
Year of diagnosis per 5‐year increments	0.85 (0.75, 0.96)	0.0096	1.06 (0.91, 1.25)	0.4598	1.07 (0.91, 1.25)	0.4379	0.65 (0.51, 0.82)	0.0004	0.88 (0.67, 1.15)	0.3413	0.84 (0.88, 1.74)	0.0353	0.93 (0.65, 1.31)	0.6629

Abbreviations: CI, confidence interval; HR, hazard ratio; ID, insufficient data; km, kilometers.

#### 3.2.2 Rhabdomyosarcoma

The 5‐year OS rate was 71.0% (95% CI 67.1%, 75.2%) for patients with Rhabdomyosarcoma. The incidence of recurrence was second highest in patients with rhabdomyosarcoma, at 36.6%. Most rhabdomyosarcoma primary tumors were found in the soft tissue of the lower limbs (34%). Across income quintiles, there was no significant trend in survival for patients with rhabdomyosarcoma (*p* = 0.201). However, patients with the medium‐low family income quintile had inferior OS compared to patients with the highest income quintile (HR 1.91 [95% CI 1.08, 3.37], *p* = 0.0264). Distance to treatment center, gender, and year of diagnosis did not significantly impact OS on multivariable analysis. Metastasis at diagnosis was associated with increased mortality (HR 3.95 [95% CI 2.76, 5.65], *p* < 0.0001; Table 3). Compared with primary tumors in the genital region, tumors in soft tissue (HR 2.32 [95% CI 1.36, 3.95], *p* = 0.002), head and neck (HR 2.28 [95% CI 1.27, 4.09], *p* = 0.0056), abdominal/retroperitoneal (HR 2.13 [95% CI 1.03, 4.38], *p* = 0.041), and other (HR 3.01 [95% CI 1.52, 5.97], *p* = 0.0016) locations had inferior OS.

#### 3.2.3 Osteosarcoma

The 5‐year OS rate was 66.3% (95% CI 61.8%, 71.0%) for patients with Osteosarcoma. The incidence of recurrence, defined as either relapse or progression, was highest in osteosarcoma (37.7%). Income quintile, year of diagnosis, and age did not impact OS on multivariable testing. Metastasis at diagnosis was associated with increased mortality (HR 3.37 [95% CI 2.38, 4.78], *p* < 0.0001; Table 3). Patients living 50 km to < 200 km from a treatment facility had inferior OS compared to patients living within 50 km from the treatment facility (HR 1.56 [95% CI 1.04, 2.34], *p* = 0.0299). Female patients had superior survival compared to male patients (HR 0.65 [95% CI 0.46, 0.93], *p* = 0.0167).

#### 3.2.4 Ewing Sarcoma

The 5‐year OS rate was 72.1% (95% CI 67.1%, 77.5%) for patients with Ewing Sarcoma. Most Ewing Sarcoma primary tumors were found in the abdomen and retroperitoneum (78%). Income quintile and distance to treatment center did not impact OS on multivariable analysis. Metastasis at diagnosis was associated with increased mortality (HR 3.35 [95% CI 2.11, 5.32], *p* < 0.0001; Table 3). Survival rates improved over time (HR 0.65 [95% CI 0.51, 0.82], *p* = 0.0004, expressed per 5‐year increase).

#### 3.2.5 Hepatoblastoma

The 5‐year OS rate was 80.1% (95% CI 75.4%, 85.1%) for patients with hepatoblastoma. The incidence of metastasis at diagnosis was lowest in hepatoblastoma (21.8%). Income quintile did not significantly impact OS in the multivariable model. Patients living > 500 km from a treatment facility had inferior OS compared to patients living within 50 km from the treatment facility (HR 3.29 [95% CI 1.40, 7.79], *p* = 0.0065). Metastasis at diagnosis was associated with increased mortality (HR 4.41 [95% CI 2.38, 8.16], *p* < 0.0001).

### 3.3 Multivariable Analysis—Recurrence With Death as a Competing Risk

#### 3.3.1 Wilms Tumor

The 5‐year cumulative incidence rate was 18.1% (95% CI 15.7%, 21.0%) for Wilms tumor. Income quintile and distance to treatment center did not impact recurrence rates in the multivariable model. Metastasis at diagnosis was associated with increased recurrence rates (HR 2.05 [95% CI 1.43, 2.96], *p* < 0.0001). Survival rate improved over time (HR 0.84 [95% CI 0.88, 1.74], *p* = 0.0353, expressed per 5‐year increase).

#### 3.3.2 Malignant Gonadal Germ Cell Tumors

The 5‐year cumulative incidence rate was 13.4% (95% CI 9.61%, 18.7%) for patients with GCTs. The incidence of recurrence, defined as either relapse or progression, was lowest in GCTs (13.8%). Income quintile and distance to treatment center did not impact recurrence on multivariable modeling.

## 4 Discussion

In this analysis of Canadian children with solid extracranial tumors, our results do not demonstrate a consistent relationship between SES or distance and survival outcomes. These results suggest that, in general, the universal Canadian health care system with robust social supports may mitigate some of the disparities in survival outcomes due to differences in SDOH noted in other settings. In Canada, frontline medical therapy for the children diagnosed with these tumors is well‐standardized and homogenous. However, there were significant findings in some groups that warrant consideration. For example, patients with neuroblastoma and rhabdomyosarcoma with highest family income quintile had superior survival than those in a lower income quintile. Yet, income did not significantly impact survival in Ewing sarcoma, hepatoblastoma, osteosarcoma, or Wilms tumor. Regarding distance, we found that patients with hepatoblastoma living furthest away (> 500 km) from a treatment facility had inferior survival compared to patients living within 50 km of the treatment facility.

Several results from this multicenter analysis supported existing literature in the field of pediatric oncology. Specifically, metastasis is a well‐documented high‐risk disease feature that impacts survival in children with solid tumors [18, 19, 20]. In terms of tumor location, our data is consistent that rhabdomyosarcoma and neuroblastoma primary tumor locations influence disease outcome [21, 22, 23]. Our data also supports age as a prognostic factor for many solid tumor diagnoses including Ewing Sarcoma [24] and neuroblastoma [25, 26, 27]. These data support the validity of new associations found with other variables. Our study spans a 19‐year period, during which therapies have evolved significantly for the included diagnoses. Survival rates for patients diagnosed with neuroblastoma, Ewing sarcoma, and Wilms tumor improved over time, highlighting efficacious treatment advancements for these tumors over this period.

In this study, we used income quintile as a surrogate measure for economic stability, a well‐known SDoH that contributes to SES [28]. In the United States, survival in children with cancer was found to be impacted by SES [28, 29]. Literature in the context of universal healthcare systems has found that lower SES did not significantly impact pediatric cancer survival outcomes in Canada [12, 13], Finland [30], or Denmark [31]. The results of our multivariable analysis suggest that overall, SES does not impact survival outcomes in solid extracranial tumors in Canada. Patients with the highest family income quintile had superior survival outcomes in neuroblastoma and rhabdomyosarcoma. These results are significant, but we did not observe a trend in survival outcomes across income quintiles. Neuroblastoma and rhabdomyosarcoma utilize different modes of treatment with well‐established systematic therapy protocols that all patients are entitled to in Canada. Some factors which may influence inferior survival with lower SES include decreased clinical trial enrollment for children with lower income, poor treatment adherence, fewer social resources, and communication barriers with healthcare providers [32, 33, 34]. A recent CYP‐C population‐based study found that children with cancer in the lowest income quintile enrolled less frequently in Phase 1 and 2 clinical trials nearing end of life [35]. Unfortunately, data on enrolment in clinical trials was not available through CYP‐C, so we were not able to assess this. Additionally, there may be tumor‐specific risk factors or other aspects of SES, like parental education, that contribute to these differences in survival in neuroblastoma and rhabdomyosarcoma that are not accounted for in our model. Further research should be focused on neuroblastoma and rhabdomyosarcoma and the impact of additional SES factors which may contribute to childhood survival, like parental education levels and employment status.

Distance to treatment center is an important factor which has the potential to impact access to health care [28]. Greater distance to tertiary care facility did not elicit a trend in survival among all solid tumors included in our multivariable analysis. Only patients with hepatoblastoma living furthest away (> 500 km from treatment facility) had inferior overall survival compared to patients living closest (< 50 km). Considering this isolated finding in the absence of a significant trend among the distance categories, this finding may be spurious. If genuinely significant, there are potential factors which may be contributing to this difference including access to clinical trial enrollment, as some studies have demonstrated superior survival among patients enrolled in therapeutic clinical trials [36]. A recent population‐based study in Canada found that patients living closer to a tertiary care center had higher clinical trial enrollments [37]. Additionally, previous studies in the USA have identified racial disparities among pediatric patients with hepatoblastoma [38, 39]. Future research should concentrate on this difference in survival for patients with hepatoblastoma living farthest away from treatment facility, with particular focus on racial disparities in a Canadian context, clinical trial availability and enrollment.

Although the results of our study are strengthened by the multivariable analysis and large sample size, several limitations should be considered. One limitation was the inability to examine the interaction between distance and income due to low frequency counts and consequently low power in some disease groups. Additionally, we collected information on neighborhood income quintile at the time of diagnosis to account for wealth, but this does not encompass changes to family income quintile over the study period (2001–2019). Moreover, other critical variables like parental education and employment were not available in CYP‐C to include in our models. It is pertinent to acknowledge that income quintile alone does not comprehensively characterize SES. Additionally, the use of neighborhood income quintile is limited by geospatial methods, making the results vulnerable to ecologic fallacy [40]. As with all large database studies, our analyses were limited by what was reliably available in CYP‐C. Most notably, we could not analyze the effects of race and ethnicity or time to seeking care on survival outcomes. These variables are considerations that should be examined in patients with neuroblastoma, rhabdomyosarcoma, and hepatoblastoma, where significant survival differences were demonstrated with lower income and farther distance.

Overall, we highlight that income quintile and distance to treatment center do not significantly impact survival outcomes in children with solid extracranial tumors in Canada. Highest family income quintile was associated with superior survival outcomes in children with neuroblastoma and rhabdomyosarcoma. Patients with hepatoblastoma living furthest away from a tertiary care facility had worse overall survival than patients living closest. However, there was no general trend across tumor groups and within distance and income variable categories. This study provides an important step in understanding the impact of SES on survival outcomes in pediatric extracranial solid tumors in Canada. While other countries have identified both distance and income to influence survival outcomes in childhood cancer, our study highlights that these differences may be mitigated by the universal healthcare system and supports offered in Canada. Additional population‐based studies using individual‐level metrics of SES are encouraged to provide further insight into the impact of distance and income on survival for pediatric solid tumor diagnoses.

## Author Contributions

**Olivia Piccolo:** conceptualization (equal), data curation (equal), formal analysis (supporting), funding acquisition (lead), investigation (lead), methodology (equal), project administration (equal), writing – original draft (lead), writing – review and editing (lead). **Kara Matheson:** data curation (equal), formal analysis (lead), methodology (equal), validation (lead), writing – review and editing (supporting). **Stacey Marjerrison:** supervision (supporting), writing – review and editing (supporting). **Ketan Kulkarni:** supervision (supporting), writing – review and editing (supporting). **Rodrigo Romao:** conceptualization (equal), supervision (supporting), writing – review and editing (supporting). **Craig Erker:** conceptualization (equal), data curation (equal), formal analysis (equal), funding acquisition (equal), investigation (equal), methodology (lead), project administration (equal), supervision (lead), writing – original draft (equal), writing – review and editing (equal).

## Disclosure

Data used in this publication are from the Cancer in Young People in Canada Surveillance Program and are used with the permission of the Public Health Agency of Canada. The analyses and interpretation presented in this work do not necessarily reflect the opinions of the federal government of Canada.

## Ethics Statement

REB approval was granted by the IWK (file no. 1029288) in July 2023.

## Conflicts of Interest

The authors declare no conflicts of interest.

## Data Availability

The data that support the findings of this study are available from CYP‐C. Restrictions apply to the availability of these data, which were used under license for this study. Data are available from Olivia Piccolo and Craig Erker with the permission of CYP‐C.
